# Supplementary material for: Characterization of the Secretome of a Specific Cell Expressing Mutant Methionyl-tRNA Synthetase in Co-Culture Using Click Chemistry
Source: Int J Mol Sci. 2022 Jun 10;23(12):6527. doi: 10.3390/ijms23126527 (PMC9223471; doi:10.3390/ijms23126527)
Supplement: Supplementary file 1 [file ijms-23-06527-s001.zip › Supplementary Figures.pdf]

Characterization of the secretome of a specific cell expressing mutant methionyl-tRNA synthetase in co-culture using click chemistry

Supplementary Table

Table S1. Protein profile of the MSC secretomes

The table is provided separately as an EXCEL spreadsheet.

Supplementary Figures

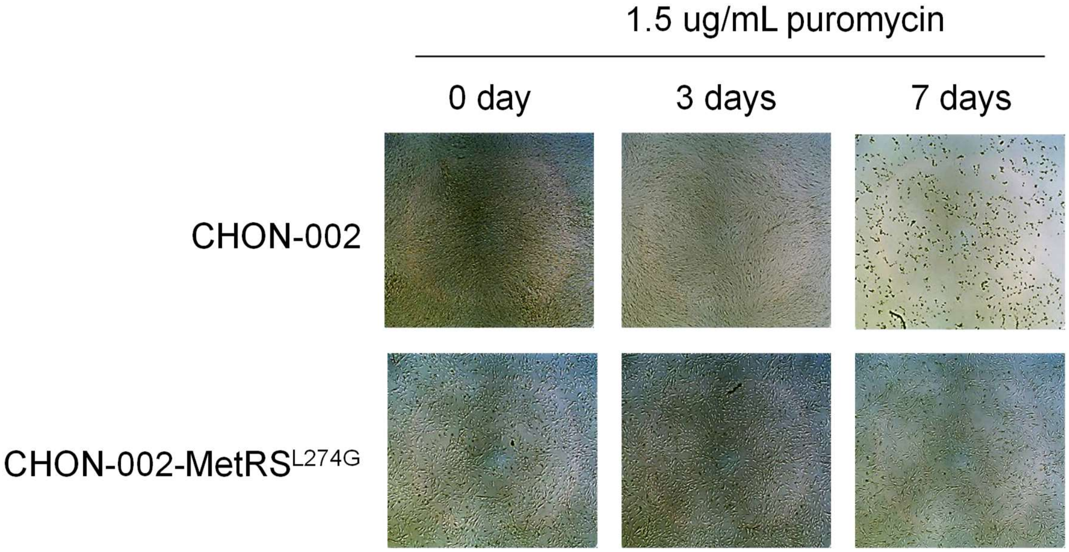

Figure S1. Selection of the transformed cells. Cells transformed with the lentiviral vector were selected using a medium containing puromycin. Most of the non-transformed cells died after 7 days in a medium with puromycin. CHON-002, non-transduced human chondrocyte cell line; CHON-002-MetRSL274G, cell line transformed to express mouse mutant MetRS^{L274G}.

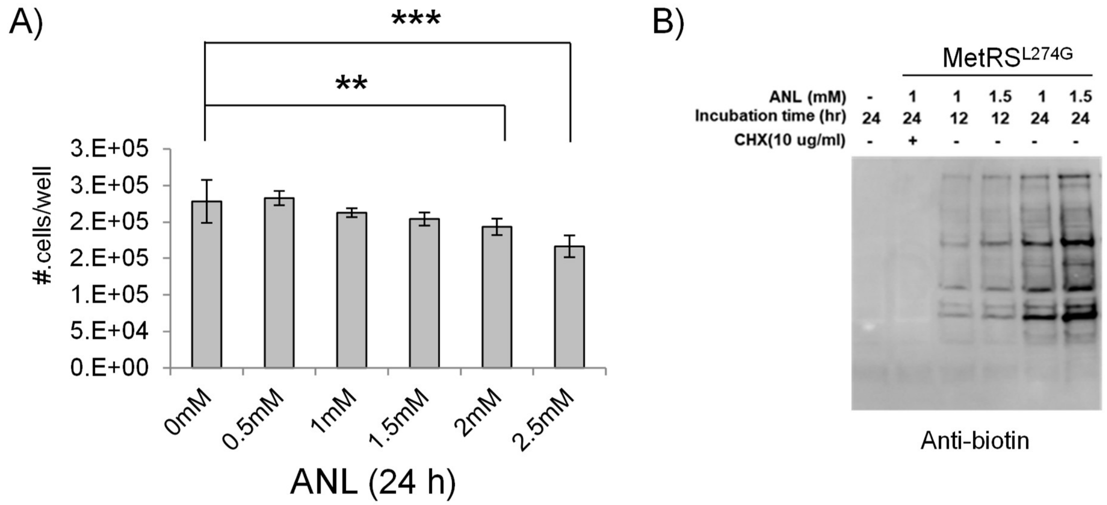

Figure S2. Optimization of cell culture conditions for protein ANL labeling. A) Cell proliferation depending on the concentrations of ANL. Cell proliferation was measured by counting the number of cells after culturing cells for 24 hours in ANL-containing medium. **, p-value < 0.05; ***, p-value < 0.01. B) Confirmation of ANL incorporation efficiency depending on the ANL concentration (1 mM or 1.5 mM) or the incubation time (12 h or 24 h). The lysate of the transduced cells cultured in ANL-supplemented media was tagged with the alkyne-activated biotinylation reagent, and the incorporation efficiency was determined based on the biotin signal in the Western blot. CHX indicates cycloheximide.

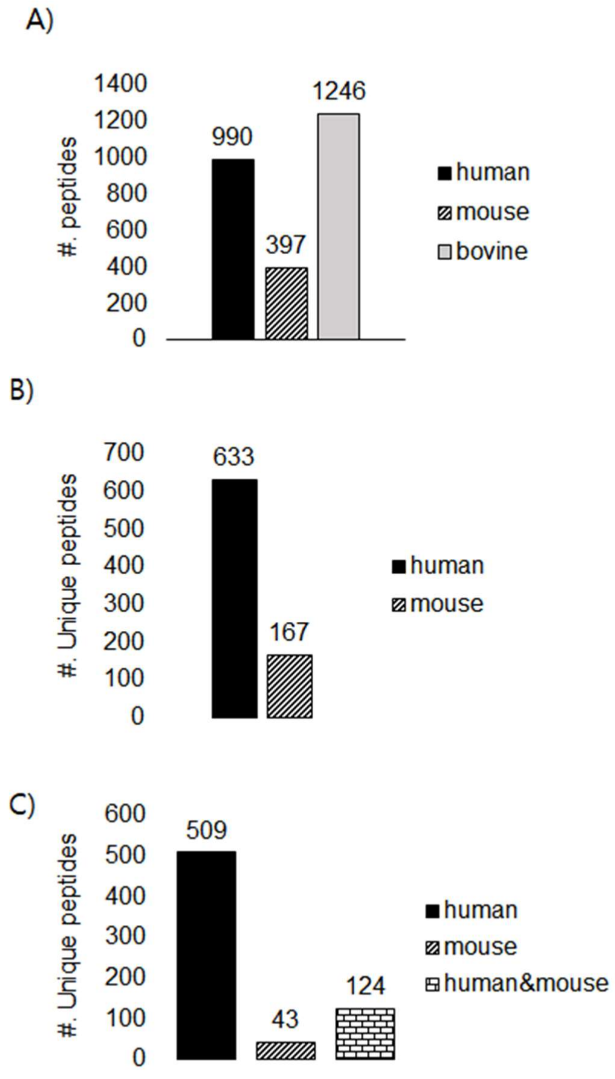

Figure S3. The number of the identified peptides in the experiment of co-culturing the transduced human cells to express the mutant MetRS and the xenogeneic mouse cells. A) The number of the identified peptides depending on the database (Uniprot human, mouse or bovine database) used in MS data search step. B) The number of human and mouse peptide groups after excluding any shared peptides with bovine proteins. C) The number of the shared peptides between human and mouse proteins (designated as human&mouse). Only unique peptides to human proteins were used for further analysis.

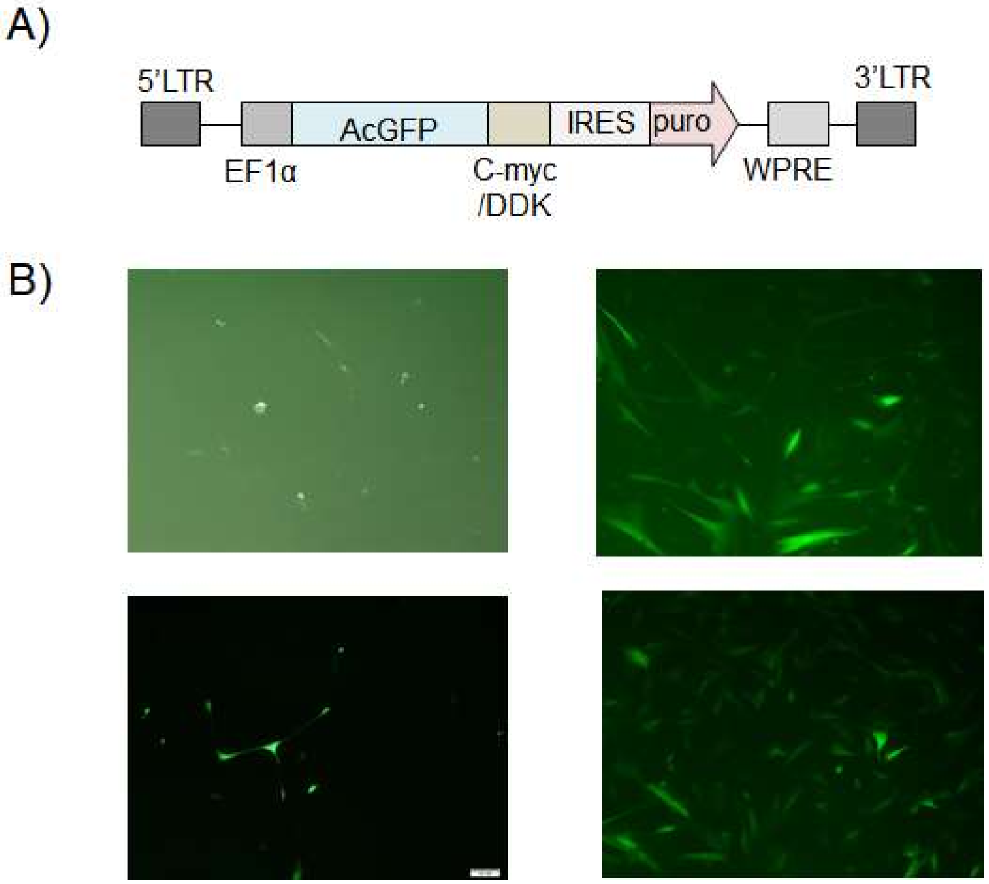

Figure S4. Confirmation of lentivirus gene expression in placenta-derived MSCs. A) Structure of GFP-expressing lentivirus vector. LTR, Long terminal repeat; EF1α, elongation factor 1 alpha; AcGFP, Aequorea coerulescens gene; DDK, FLAG tag; IRES, internal ribosome entry site; puro, puromycin resistance gene; WPRE, WHP posttranscriptional regulatory elemental. B) Analysis of GFP fluorescence in the transduced MSCs. GFP expression was measured after culturing MSCs for 24 hours.
